# Comparative analysis of microRNA expression profiles in shoot and root tissues of contrasting rice cultivars (*Oryza sativa* L.) with different salt stress tolerance

**DOI:** 10.1371/journal.pone.0286140

**Published:** 2023-05-24

**Authors:** Duc Quan Nguyen, Ngoc Lan Nguyen, Van Tung Nguyen, Thi Huong Giang Tran, Thanh Hien Nguyen, Thi Kim Lien Nguyen, Huy Hoang Nguyen

**Affiliations:** 1 Institute of Genome Research, Vietnam Academy of Science and Technology, Hanoi, Vietnam; 2 Graduate University of Science and Technology, Vietnam Academy of Science and Technology, Hanoi, Vietnam; Royal Holloway University of London, UNITED KINGDOM

## Abstract

Rice is the second-most important primary crop in the world and one of the most susceptible crops to salt stress. Soil salinization hinders seedling growth and decreases crop yield by inducing ionic and osmotic imbalances, photosynthesis disturbances, cell wall alterations, and gene expression inhibition. Plants have developed a range of defense mechanisms to adapt to salt stress. One of the most effective means is to make use of plant microRNAs (miRNAs) as post-transcriptional regulators to regulate the expression of developmental genes in order to mitigate the detrimental effects of salt stress. In this study, the miRNA sequencing data between two contrasting rice cultivars, salt-tolerant Doc Phung (DP) and salt-sensitive IR28 seedlings, were compared under control and salt stress (150 mM NaCl) conditions to determine the salt stress-responsive miRNAs. Comparative analysis of miRNA sequencing data detected a total of 69 differentially expressed miRNAs in response to salt stress treatment. Among them, 18 miRNAs from 13 gene families, *MIR156*, *MIR164*, *MIR167*, *MIR168*, *MIR171*, *MIR396*, *MIR398*, *MIR1432*, *MIR1846*, *MIR1857*, *MIR1861*, *MIR3979*, and *MIR5508*, were identified to be specifically and significantly expressed in the shoot and root tissues of DP seedlings. Gene ontology (GO) and Kyoto Encyclopedia of Genes and Genomes (KEGG) enrichment analyses further revealed that these detected miRNAs regulate a range of essential biological and stress response processes, including gene transcription, osmotic homeostasis, root formation, ROS scavenger synthesis, and auxin and abscisic acid signaling pathways. Our findings provide more insight into the miRNA-mediated responsive mechanisms of rice under salt stress and should benefit the improvement of salt stress tolerance in rice.

## Introduction

Soil salinization is a serious global problem that is known to impede the agricultural yield growth of food crops. Soil salinization is mainly caused by extreme weather, climate change, and unsustainable human farming techniques. These factors lead to the excessive accumulation of soluble salt ions in the soil, such as anions (Na^+^, Ca^2+^_,_ and Mg^2+^) and cations (Cl^-^, ClSO_3_^-,^ SO_4_^2-^, HCO_3_^-^_,_ and CO_3_^2-^) [1–3]. It has been shown that soil significantly affects the sustainable agricultural development of more than 100 countries [3, 4]. Asia has the most salt-affected agricultural land with 194.7 million hectares (ha), followed by America with 77.6 million ha, Africa with 53.5 million ha, Australia with 17.6 million ha, and Europe with 7.8 million ha [4, 5]. It is estimated that soil salinization costs the global agricultural economy more than 12 billion US dollars each year [1].

Rice is the second-most important crop in the world and is currently providing food for 7.9 billion people. Rice production is expected to meet the demand of 9.7 billion people by 2050. However, rice is known as one of the cereal crops that is most vulnerable to salt stress, particularly during seedling and reproductive stages, with most cultivated cultivars having a salinity tolerance threshold of 3.0 deciSiemens per meter (ds/m) [6, 7]. Excessive salt accumulation results in ionic imbalance, osmotic pressure, photosynthesis disturbances, and changes in cell wall structure and composition in plant tissues [8, 9]. Increased Na^+^ and Cl^-^ ion absorption also inhibits the expression of genes involved in a range of biochemical and physiological processes, including those encoding photosynthetic enzymes like RuBisCo (RIBULOSE BISPHOSPHATE CARBOXYLASE), NADP-malic, PEPC (PHOSPHOENOLPYRUVATE CARBOXYLASE), and PPDK (PYRUVATE ORTHOPHOSPHATE DIKINASE). These problems cause plant growth retardation, delayed flowering, and poor panicle development. Rice productivity has been reported to be reduced by 10% at the EC of 3.5 dS/m and by 50% at the EC of 7.2 dS/m [10–12].

Plants have evolved many adaptation mechanisms in response to salt stress [13]. The mechanisms of salinity adaptation involve a number of processes that fall into three modes: (1) osmotic adjustment to minimize osmotic stress, (2) exclusion of toxic ions to maintain ion homeostasis, and (3) compartmentalization of accumulated salt to protect plants from oxidative stress [2]. At the molecular level, these activities involve the spatio-temporal interaction of a miRNA network; the network that ultimately regulates the expression of a wide variety of genes encode transcription factors and regulator proteins associated with salinity adaptation, including NAC, MYB, MADS-box transcription factors, SALT OVERLY SENSITIVE, HIGH AFFINITY POTASSIUM TRANSPORTER, HIGH-AFFINITY NA+/K+ SYMPORTER, VACUOLAR CALCIUM ION TRANSPORTER, and Δ1-PYRROLINE-5-CARBOXYLATE-SYNTHETASE1 [13–15].

MiRNAs are small (18–25 nucleotides, nt), endogenous, non-coding, conserved RNA molecules that regulate gene expression at the post-transcriptional level in a variety of plant biological processes and stress responses [16, 17]. The identification of miRNAs associated with salt tolerance may pave the way for a more effective genetic strategy for generating salt-tolerant plants. With the advances in next-generation sequencing, profiling of the plant microRNA landscape under different experimental conditions has been routinely performed [18, 19]. To date, many miRNAs have been identified and characterized as being involved in the mechanisms of salt stress responses. Pegler and colleagues studied miRNA landscapes in *Arabidopsis* and *Setaria viridis* under salt stress conditions and revealed a more than 8.4-fold reduction in the expression level of miR397, a miRNA that controls the expression of *LACCASE* family members involved in the lignin biosynthesis pathways of monocotyledonous and dicotyledonous plants [20, 21]. In cereals, an increasing number of miRNAs associated with salt tolerance have also been identified. MiR172, for example, has been found to have a role in ionic detoxification in rice and wheat via modulating the expression of genes encoding reactive oxygen species (ROS) scavengers [22]. In addition, miR393 is important for salt-induced tillering and early flowering in rice [23]. In this study, high-throughput sequencing technology was employed to generate the miRNA profiles of the two contrasting rice cultivars, salt-tolerant Doc Phung (DP) and salt-sensitive IR28, under control and salt stress (150 mM NaCl) treatments. By comparing the miRNA profiles of DP and IR28 seedlings, the differentially expressed miRNAs (DEmiRNAs) were identified. GO and KEGG analyses were also performed to identify the cellular and metabolic pathways related to the identified DEmiRNAs and their target genes in response to salt stress in two rice cultivars. Our findings will provide a valuable resource on salt stress-responsive miRNAs that could serve as a platform for future research on the development of rice cultivars with high yields and salt tolerance.

## Material and methods

### Plant material

Two rice cultivars, *Oryza sativa* L. Doc Phung (DP, a salt-tolerant cultivar), and IR28 (a salt-sensitive cultivar), were used in this study. Seeds of DP and IR28 cultivars were provided by the Faculty of Agriculture, Can Tho University, Can Tho, Vietnam. Sixty mature seeds of each cultivar were initially dehusked and surface sterilized in 30 mL of 5% (v/v) commercial bleach (NaOCl) and 0.1% (v/v) Tween-20 for 5 minutes at room temperature. Bleached seeds were then washed with distilled water three times and soaked in water overnight to improve the germination rate and uniformity. The next day, surface sterilized seeds were air-dried on sterilized filter paper before 12 seeds were transferred onto a glass tissue culture jar containing 1/2 strength Murashige and Skoog (MS) solid medium (1/2 X MS basal salt powder, 2% sucrose, pH: 5.7) and cultivated under standard growth conditions of 16 h of light (~600 μmol.m^-2^.s^-1^)/ 8h of darkness and 24 ± 2°C. Seven days after germination, an equal number of DP and IR28 seedlings were transferred to tissue culture jars containing either fresh control medium (solid MS medium– 1X MS basal salt powder, 20% sucrose, pH: 5.7) or salt stress medium (control medium supplemented with 150 mM sodium chloride (NaCl)). The EC of 150 mM NaCl is approximately 15 dS/m. A NaCl concentration of 150 mM was chosen for salt stress treatment because it provides a clear distinction in the growth and development of DP and IR28 cultivars. Rice seedlings were maintained under the same growth conditions for an additional 7 days.

### Phenotypic and physiological analyses of DP and IR28 seedlings

Phenotypic assessments of two rice cultivars under two treatment conditions were conducted following the description of Pegler [20]. Fourteen-day-old seedlings of DP and IR28 cultivars from 2 conditions (control and salt stress treatments) were harvested for phenotypic analyses, including shoot length (from the basal node to the tip of the leaf blade), primary root length, and dry weight. All the measurements were conducted with at least three replications.

Measurement of total chlorophyll in the leaves of DP and IR28 seedlings under control and salt stress treatments was conducted according to Lichtenthaler and Wellburn [24]. The third true leaf of each seedling was harvested. The total chlorophyll content was calculated by the use of the equation: Total chlorophyll (mg/g fresh weight (FW)) = (6.43 x A663 + 18.43 x A646) / sample weight (g) x 1000. Whereas, A663 and A646 are the absorbance at 646 and 663 nanometers (nm), respectively.

### Extraction of total RNA

Total RNA from the five biological replicates of shoot and root plant material from DP and IR28 seedlings was extracted by the Ribospin^TM^ Plant Kit (GeneAll, Korea) according to the manufacturer’s instructions. The extracted RNA was then treated with DNase I (ThermoFisher Scientific, Vietnam) for the removal of DNA contamination and purified by the filter column provided by the kit. The quantification and qualification of the extracted RNA samples were assessed by gel electrophoresis on 1.2% agarose gel and using a Nanodrop^TM^ 2000 spectrophotometer (ThermoFisher Scientific, Vietnam).

### High-throughput sequencing of miRNA

One microgram (1.0 μg) of total RNA from each of the five biological replicates for each tissue sample of DP and IR28 seedlings was pooled together. Five micrograms (5 μg) of total RNA from eight samples, including DP control shoots, DP control roots, IR28 control shoots, IR28 control roots, DP salt-stressed shoots, DP salt-stressed roots, IR28 salt-stressed shoots, and IR28 salt-stressed roots, were used for sequencing. The library was constructed using the TruSeq Small RNA Library Preparation Kit (Illumina, USA) according to the manufacturer’s instructions prior to being used for miRNA sequencing (150 paired-end and 20 million reads) on the Illumina HiSeq system.

### Analysis of miRNA sequencing data

Post miRNA sequencing, raw sRNA reads were processed to remove the 3’ end-adapter sequences and filtered to remove the reads that have an average quality score lower than 20 and shorter than 18 bp using Trimmomatic tool (v0.40; RRID: SCR_011848). The filtered reads were then aligned to the miRbase database (release 22.1) using the bowtie2 alignment tool (v2.1.0; RRID: SCR_016368). Perfectly aligned reads were then used for microRNA annotation. The miRNA data was expressed as an expression of transcripts per million (TPM). The members of each miRNA family with an identical mature sequence were counted as a single miRNA. The total number of miRNA raw reads per library, the total number of high quality raw reads per library, the values of each miRNA read count across control and salt stress treatments, and the Log_2_ fold change in abundance between control and salt stress treatments were determined. Log_2_ fold change is calculated using the equation of log_2_ (normalized expression of miRNA (TPM) under salt stress/ normalized expression of miRNA (TPM) in control condition).

A Venn diagram and Heatmap were constructed to determine the distribution and profiles of detected miRNAs in shoot and root tissues of DP and IR28 under control and salt stress conditions, respectively.

### Synthesis of complementary DNA

One microgram (1.0 μg) of high-quality RNA from each plant sample was used as a template for the synthesis of cDNA using the ProtoScript^®^ First Strand cDNA Synthesis Kit (New England Biolabs, USA), according to the manufacturer’s instructions. A miRNA-specific cDNA was synthesized from 300 ng of total RNA using 50 U of ProtoScript^®^ reverse transcriptase and 50 nM of miRNA-specific stem-loop primer. This procedure was also performed for the synthesis of reference genes, U6. The primers used for the synthesis of miRNA-specific cDNA are listed in S1 Table.

### Quantitative reverse transcriptase polymerase chain reaction analysis

A RT-qPCR analysis was used to evaluate the abundance of mature miRNAs and the expression level of their target genes in samples of DP and IR28 cultivars under control and salt stress treatments. For validation of the miRNA sequencing data, six miRNAs, miR164d, miR169i, miR172d, miR398a, miR528, miR529b and their target genes, *NAC21/22* (*LOC_Os06g46270*), *NUCLEAR TRANSCRIPTION FACTOR Y SUBUNITS* (*NTFY*; *LOC_Os03g44540*), *AP2-LIKE ETHYLENE-RESPONSIVE TRANSCRIPTION FACTORS* (*AP2/ERF*; *LOC_Os03g60430*), *SUPEROXIDE DISMUTASE [CU-ZN] 2-LIKE* (*CSD2*; *LOC_Os08g44770*), *L-ASCORBATE OXIDASE* (*AO*; *LOC_Os06g37150*), and *SQUAMOSA PROMOTER-BINDING PROTEIN-LIKE 14 (SPL14; LOC_Os08g39890*) respectively, were selected. RT-qPCRs were carried on LightCycler® 96 System (Roche, USA). Each reaction (10 μL) was prepared using Luna® Universal qPCR Master Mix (NEB, USA) according tothe manufacturer’s instructions. The two housekeeping genes, *ACTIN* (*ACT*; *LOC_Os03g50885)* and *UBIQUITIN* (*UBQ; LOC_Os02g06640*), were used as reference genes [13]. The primers used for RT-qPCR are listed in S1 Table. RT-qPCR analysis of each gene was conducted with four biological replications and three technical replications.

### GO and KEGG enrichment analysis

Gene ontology (GO) enrichment analysis was conducted to annotate the molecular function, cellular components, and biological processes of miRNA target genes using the GOSeq (v1.34.1; RRID: SCR_017052) tool.

KEGG enrichment analysis was performed by blasting against the KEGG database (https://www.genome.jp/kegg/) to determine the biological pathway of miRNA target genes.

### Statistical analysis

Statistical analysis was conducted by a t-test or One-way ANOVA (RRID: SCR_002427) and Tukey’s *post hoc* with at least three biological replicates. The statistical significance was represented by an asterisk or a different letter on each histogram (*p* ≤ 0.05).

## Results

### Phenotypic and physiological assessment of DP and IR28 seedlings under control and salt stress conditions

**Fig 1:**
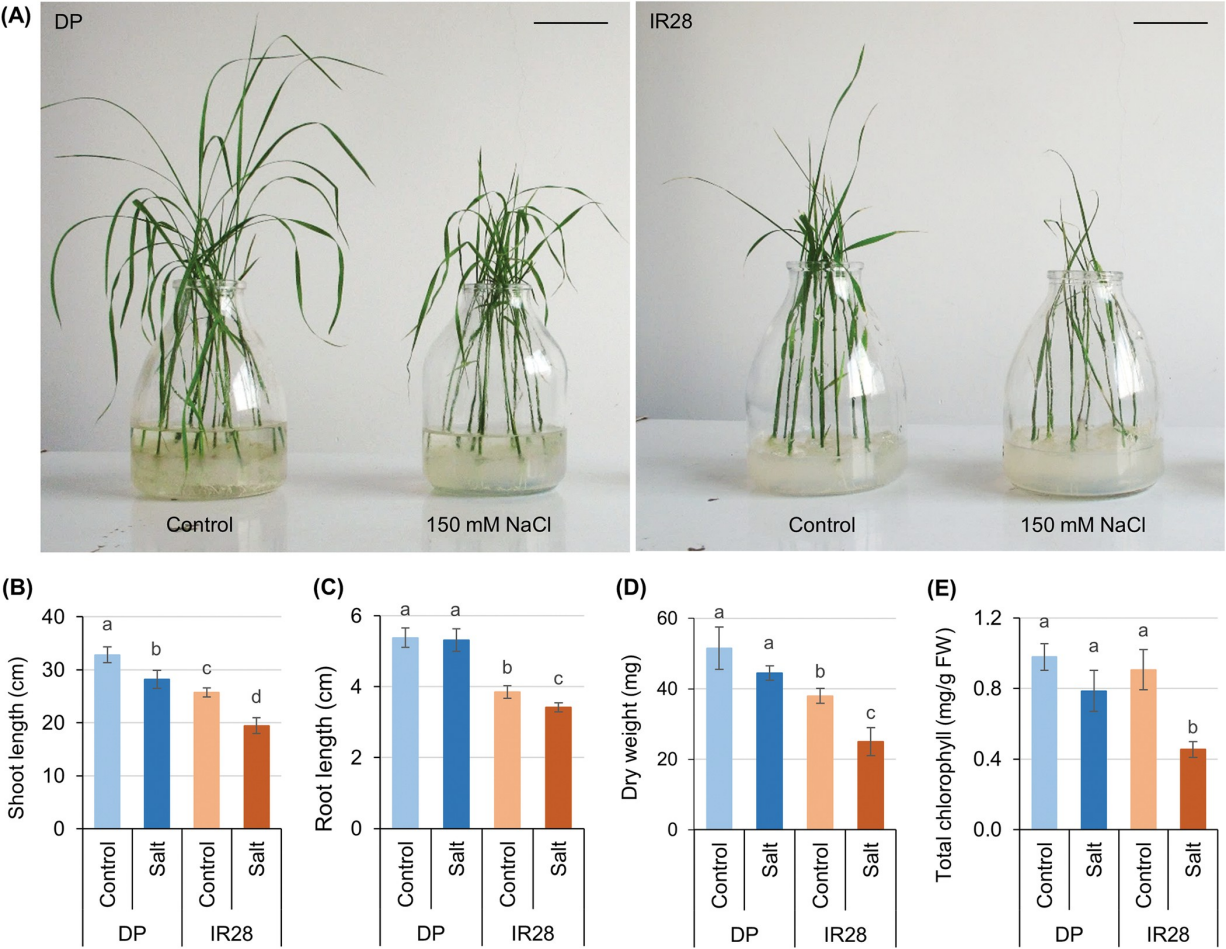
Phenotypic and physiological assessment of DP and IR28 seedlings under control and salt stress conditions. (A) Phenotypes of DP and IR28 seedlings under two experimental conditions. Bar = 5 cm. Comparison of (B) shoot length (cm), (C) root length (cm), (D) whole seedling dry weight (mg), and (E) total chlorophyll of DP and IR28 seedlings under control and salt stress treatments.

Fourteen-day-old rice seedlings were harvested for phenotypic and physiological analyses after cultivating under standard (control) and salt stress conditions. Phenotypic analysis revealed that salt stress stunted the vegetative development of salt-tolerant DP and salt-sensitive IR28 seedlings at different degrees (Fig 1A). The shoot length of DP seedlings was significantly reduced by 14.1%, from 32.8 to 28.2 cm. This reduction was approximately 24.4% in IR28 seedlings, with the shoot length decreasing from 25.7 to 19.4 cm (Fig 1B). IR28 seedlings also appeared to have a thinner stem and a higher number of wilted and rolled leaves. As expected, the dry weight of IR28 seedlings was reduced to a greater level (34.2%) than that of DP seedlings (13.6%) (Fig 1D). Salt stress has also been found to impair the photosynthesis of DP and IR28 seedlings. The total chlorophyll content of salt-stressed DP and IR28 seedlings has been found to decrease by approximately 20% and 50%, respectively (Fig 1E). Interestingly, salt stress had little effect on the primary root length of DP seedlings (a reduction of 1.2%), in comparison to an approximately 11.3% reduction in the length of the IR28 root system (Fig 1C). The phenotypic and physiological assessment results clearly demonstrated that salt stress treatment at 150 mM NaCl was highly detrimental to the growth and development of IR28 seedlings, a salt-sensitive cultivar, while this salt stress treatment only relatively slowed the development of salt-tolerant DP seedlings.

### Analysis of miRNA sequencing data

Eight miRNA libraries of DP and IR28 (IR) were generated for high-throughput sequencing. They were produced from eight sample groups, comprising control groups of shoots (CS), control groups of roots (CR), salt-treated groups of shoots (NS), and salt-treated groups of roots (NR). A total of 8.5 billion reads were obtained, including 21,760,361 from the DPCS library, 21,050,878 from the DPCR library, 21,715,247 from the DPNS library, 21,129,744 from the DPNR library, 21,687,890 from the IRCS library, 21,070,517 from the IRCR library, 21,059,869 from the IRNS library, and 20,601,489 from the IRNR library. A summary of clean reads obtained from each library after data trimming by the Trimmomatic tool is provided in Table 1.

**Table 1:** Summary of miRNA libraries of DP and IR28 seedlings under control and salt stress conditions.

Library ID	Tissue	Cultivar	Growth condition	Known miRNAs	Clean reads
DPCS	Shoot	Doc Phung	Control	156	8,505,871
DPCR	Root	Doc Phung	Control	161	9,717,054
DPNS	Shoot	Doc Phung	Salt stress	144	9,430,191
DPNR	Root	Doc Phung	Salt stress	150	9,380,216
IRCS	Shoot	IR28	Control	163	10,797,960
IRCR	Root	IR28	Control	159	11,368,926
IRNS	Shoot	IR28	Salt stress	154	11,215,734
IRNR	Root	IR28	Salt stress	153	9,234,880

A total of 615 mature miRNAs have been detected from the obtained sequencing data. High-throughput sequencing of small RNAs (sRNAs) yielded the identification of 224 known miRNAs; the miRNAs that their mature sequences are homologous to those of miRNAs published in the miRbase database (S2 Table). The total number of known miRNAs in the shoot and root tissues of both rice cultivars varied and appeared to be slightly decreased under salt stress (Table 1).

Among the 224 known miRNAs, 71 conserved miRNAs were found to belong to 20 highly conserved miRNA gene families, including the *MIR166* gene family, which has 12 members and is the largest miRNA family; the *MIR156* and *MIR167* gene families, which have 11 and 10 members, respectively; the *MIR160* and *MIR171* gene families, which have 6 and 5 members, respectively; the *MIR164* and *MIR396* gene families, which each have 4 members; the *MIR162*, *MIR169*, *MIR172*, *MIR393*, *MIR398*, and *MIR529* gene families, which each have 2 members; and *MIR159*, *MIR168*, *MIR390*, *MIR394*, *MIR408*, *MIR528*, and *MIR535* gene families, which each have only a single member.

**Fig 2:**
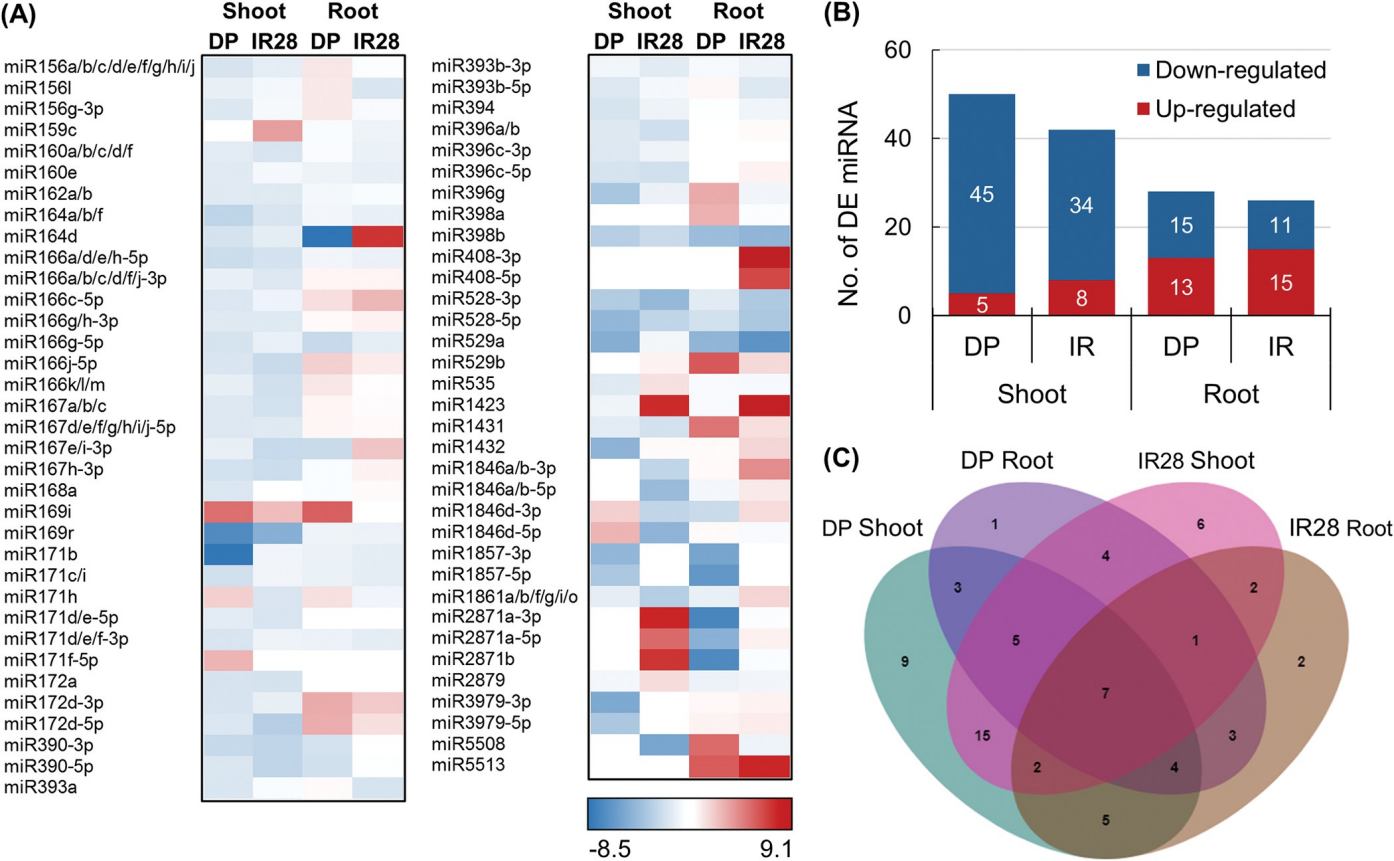
Profiling of miRNAs in the shoot and root tissues of DP and IR28 seedlings under salt stress. (A) Heat-map represents the Log_2_ fold change in abundance of miRNAs detected via high throughput sequencing. (B) Number of differentially expressed miRNAs in the shoot and root tissues of DP and IR28 seedlings. (C) Venn diagram representing the distribution of miRNAs in shoot and root tissues of DP and IR28 seedlings under salt stress.

Under salt stress, the expression of detected miRNAs in the shoot and root tissues of the two rice cultivars differed significantly (Fig 2A). The Heatmap depicted the expression profile of 69 differentially expressed miRNAs (DEmiRNAs), defined as those miRNAs with at least a 2-fold change in abundance in response to salt stress and a false discovery rate (FDR) of less than 0.05 (S3 Table). Under salt stress, 59 miRNAs were identified as DEmiRNAs in DP seedlings, while 56 miRNAs were identified as DEmiRNAs in IR28 seedlings (Fig 2B). In particular, salt stress induced the upregulation of 5 and 13 miRNAs and the downregulation of 45 and 15 miRNAs in the shoot and root tissues of DP seedlings, respectively. The most upregulated transcript abundance in DP seedlings was miR169i (5.2-fold) in shoot tissues and miR529b (6.0-fold) in root tissues. The greatest downregulated transcript abundance was miR171b (-8.3-fold) in shoots and miR164d (-8.5-fold) in roots. In IR28 seedlings, the transcript abundance in the shoot and root tissues was found to be upregulated in 8 and 15 miRNAs and downregulated in 34 and 11 miRNAs, respectively. The most increased transcripts were miR2871a-3p (7.9-fold) in shoots and miR408-3p (9.1-fold) in roots. The most decreased transcripts were miR5508 (-5.5-fold) in shoot tissues and miR529a (-6.6-fold) in root tissues.

The Venn diagram analysis further revealed the miRNAs that are uniquely expressed in the shoot and root tissues of DP and IR28 seedlings (Fig 2C). In particular, 9 miRNAs were detected in shoot tissues of DP seedlings (miR156g-3p, miR160e, miR167h-3p, miR168a, miR171f-5p, miR394, miR396c-3p, miR3979-3p, miR3979-5p) and 6 miRNAs in shoot tissues of IR28 seedlings (miR159c, miR166a/b/c/d/f/j-3p, miR166k/l/m, miR393b-3p, miR1846a/b-5p, miR2879), while only one (miR398a) and two (miR408-5p, miR408-3p) miRNAs were specifically expressed in root tissues of DP and IR28 seedlings, respectively. Three (miR396g, miR1857-3p, miR1857-5p) and two (miR1423, miR1846a/b-3p) miRNAs were detected to be expressed in the shoot and root tissues of DP and IR28 seedlings, respectively. Seven miRNAs, including miR164d, miR172d-5p, miR398b, miR528-5p, miR528-3p, miR1431, and miR1846d-3p, have been detected to be expressed across all libraries (Fig 2C).

### Prediction of target genes of differently expressed miRNAs

**Fig 3:**
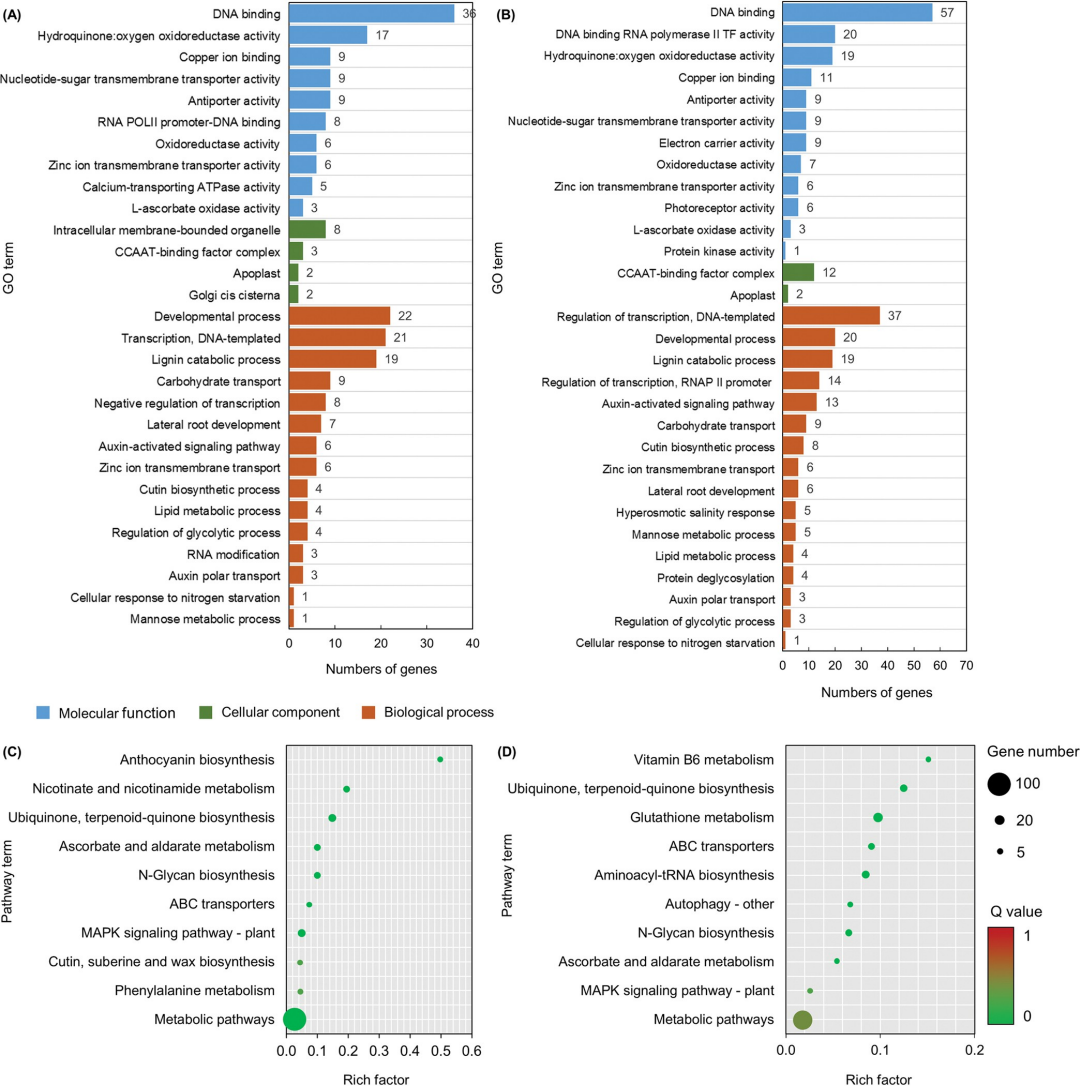
Gene ontology (GO) and Kyoto Encyclopedia of Genes and Genomes (KEGG) enrichment analysis of differently expressed miRNA targets in DP and IR28 seedlings. GO analysis revealed that miRNA targets are involved in 29 and 30 processes in DP (A) and IR28 (B) seedlings, respectively. KEGG enrichment analysis revealed the top 10 enriched pathways in DP (C) and IR28 (D) seedlings. The rich factor represents the degree of enrichment. The color code is to indicate different Q value ranges, with the smaller the Q value representing the more significant the enrichment.

The target genes for the 69 DEmiRNAs were identified using the TargetFinder (v1.7), psRNATarget prediction tools, and the TarDB sRNA-seq online database [25, 26]. There were a total of 120 predicted targets for 59 DEmiRNAs in the DP seedlings and 109 predicted targets for 56 DEmiRNAs in the IR28 seedlings (Fig 3A and 3B). Many DEmiRNA have been found to target genes encoding stress-responsive transcription factors, including members of the NAC domain-containing proteins, AP2/ERF, SPLs, NTFY, HOMEOBOX-LEUCINE ZIPPER PROTEINS (HD-zip), MYB, and MADS-box transcription factors. The DEmiRNA targets were also found to encode a variety of other proteins, such as L-ASCORBATE OXIDASES, MULTICOPPER OXIDASES, SUPEROXIDE DISMUTASE-LIKE, LACCASES, F-BOX PROTEINS, AUXIN EFFLUX CARRIERS, ZINC TRANSPORTERS, CALCIUM TRANSPORTERS, and ABC TRANSPORTERS. GO enrichment analysis was then used to annotate their biological functions. Target genes of DP and IR28 seedlings were highly enriched in 29 and 30 GO terms (*p* value ≤ 0.05), respectively (Fig 3A and 3B). For molecular function, DNA binding and hydoquinone:oxygen oxidoreductase activity were among the most enriched terms. For the cellular component, the common terms included CCAAT-binding factor complex and apoplast. For the biological process, the top three enriched terms were developmental process, regulation of transcription, and lignin catabolic process.

**Fig 4:**
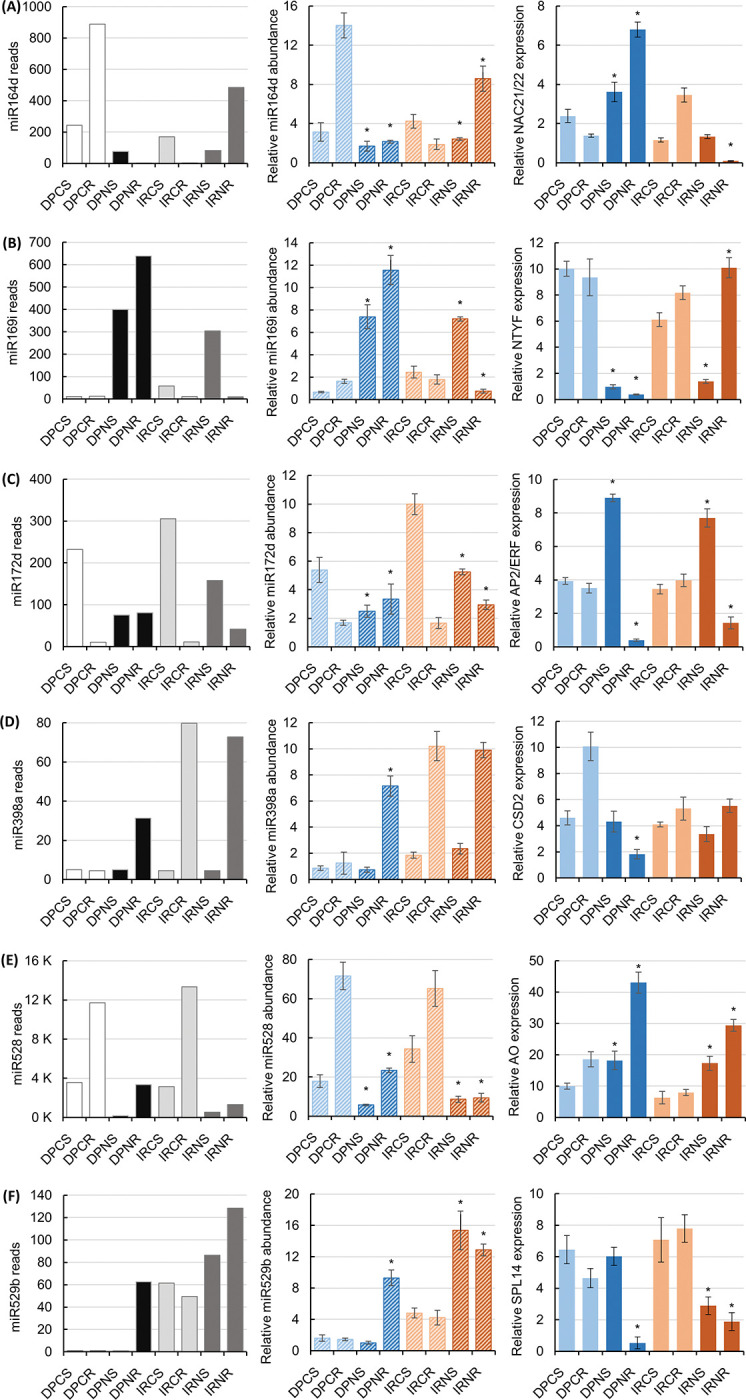
Experimental validation of selected miRNAs and molecular assessment of their targets in DP and IR28 seedlings under control and salt stress. RT-qPCR quantification of miR164d abundance and its target gene expression, *NAC21/22* (A), miR169i abundance and *NTFY* expression (B), miR172d abundance and *AP2/ERF* expression (C), miR398a abundance and *CSD2* expression (D), miR528 abundance and *AO* expression (E), and miR529b and *SPL14* expression (F). Statistical data was analyzed by a t-test and the statistically significant difference of shoot and root samples between two experimental conditions in each cultivar was presented by an asterisk (*p* < 0.05).

Target genes were subjected to a KEGG enrichment analysis to determine the molecular pathway involved in salt stress responses in DP and IR28 seedlings. Target genes of DP and IR28 seedlings were shown to participate in 90 and 84 pathways, respectively. The top ten significantly enriched pathways (*p* value ≤ 0.05) are presented in Fig 4C and 4D. These pathways are considered to play a critical role in the salt stress responses in both rice cultivars. Six out of ten pathways were both found in DP and IR28 seedlings, including ubiquinone, terpenoid-quinone biosynthesis (ko00130), ascorbate and aldarate metabolism (ko00053), N-glycan biosynthesis (ko00510), ABC transporters (ko02010), the MAPK signaling pathway–plant (ko04016), and the metabolic pathway (ko01100). Whereas, target genes involved in anthocyanin biosynthesis (ko00942), nicotinate and nicotinamide metabolism (ko00760), cutin, suberin and wax biosynthesis (ko00073), and phenylalanine metabolism (ko00360) were found to be highly enriched in DP seedlings. IR28 target genes were involved in the pathways of vitamin B6 metabolism (ko00750), glutathione metabolism (ko00480), aminoacyl-tRNA biosynthesis (ko00970), and autophagy–other processes (ko04136). These data suggest that the responses of DP and IR28 seedlings to salt stress were partially different.

### Experimental validation of miRNA sequencing data

To validate the miRNA sequencing data, a RT-qPCR analysis was conducted to evaluate the abundance of the six randomly selected DEmiRNAs and the expression of their target genes. The selected miRNAs were miR164d, miR169i, miR172d, miR398a, miR528, and miR529b. They have been identified from previous research and predicted by TargetFinder and psRNATarget tools to target genes encoding NAC21/22 (*LOC_Os06g46270*), NTFY (*LOC_Os03g44540*), AP2/ERF (*LOC_Os03g60430*), CSD2 (*LOC_Os08g44770*), AO (*LOC_Os06g37150*), and SPL14 (*LOC_Os08g39890*; S1 Fig). These target genes are involved in diverse cellular activities, including DNA binding, transcriptional regulation, electron transfer, ROS homeostasis, and cell wall thickening [22, 27–29].

Fig 4 revealed that the abundance of the selected miR164d, miR169i, miR172d, miR398a, miR528 and miR529b derived from the RT-qPCR analysis was consistent with the obtained miRNA sequencing data. Particularly, salt stress significantly decreased the abundance of miR164d and miR528 in the shoot (-1.9-fold and -3.1-fold, respectively) and root (-6.5-fold and -3.2-fold, respectively) tissues of DP seedlings. In contrast, salt stress significantly increased the abundance of miR169i in the shoot (11.2-fold) and root (7.1-fold) tissues of DP seedlings. An opposing trend was found in the abundance of miR172d. The miR172d transcript was downregulated by 2.2-fold in the shoot tissues, whereas it was upregulated by 2.0-fold in the root tissues of salt-stressed DP seedlings. The abundance of the miR398a and miR529b transcripts was slightly reduced (-0.9-fold and -0.6-fold, respectively) in the salt-stressed shoot tissues, but it was increased (5.7-fold and 6.5-fold, respectively) in the salt-stressed root tissues (Fig 4). In IR28 seedlings, the abundance of miR164d, miR169i, miR172d, and miR528 in the shoot tissues has shown a similar expression pattern to that of the corresponding miRNAs in the shoot tissues of DP seedlings under salt stress. Specifically, a decrease in abundance was found in miR164d (-1.8-fold), miR172d (-1.9-fold), and miR528 (-4.0-fold). An increase was found in the abundance of miR169i (2.9-fold) and miR529b (3.2-fold) in response to salt stress. In the salt-stressed root tissues, the abundance of miR164d, miR172d, and miR529b was greatly increased by 4.6-,1.8-, and 3.0-fold, respectively. Whereas the abundance of miR169i and miR528 was considerably decreased by 2.4- and 6.9-fold under salt stress, respectively. Interestingly, the abundance of miR398a transcript in shoot and root tissues was not significantly changed under salt stress. Next, RT-qPCR analysis was performed to evaluate the expression of miRNA target genes under salt stress. RT-qPCR data showed that the expression of genes encoding NAC21/22, NTFY, AP2/ERF, CSD2, AO, and SPL14 in the shoot and root tissues of salt-stressed DP and IR28 seedlings exhibited an opposing trend in response to the changes in abundance of miR164d, miR169i, miR172d, miR398a, miR528, and miR529b, respectively (Fig 4). Together, the results presented in Fig 4 have validated the accuracy of the miRNA sequencing data as well as the suitability of using either miRNA sequencing or RT-qPCR approaches in the generation of an accurate expression profile of miRNAs in DP and IR28 seedlings under salt stress.

## Discussion

Salt stress is an abiotic stress that severely inhibits the growth, development, and productivity of numerous agricultural crops, especially during the early stages of development [2, 3, 14, 30]. Crop plants have developed a range of strategies in response to the increasing concentration of Na^+^ and Cl^-^ ions in soil [1]. The post-transcriptional regulation of developmental and stress-responsive genes by miRNAs has been considered one of the most effective strategies against salt stress. In this study, we profiled the miRNA expression landscape of the salt-tolerant DP and salt-sensitive IR28 seedlings using high-throughput sequencing and compared the obtained sequencing data to acquire a better understanding of the regulatory activities of miRNAs in response to salt stress.

Firstly, phenotypic and physiological analyses were conducted to determine the impacts of salt stress on the vegetative development of DP and IR28 seedlings at the early stage of plant development. Cultivation of 7-day-old DP and IR28 seedlings for a period of seven days on half-strength MS media supplemented with 150 mM NaCl reduced their growth and vegetative development. For most crop plants, the accumulation of a high concentration of Na^+^ and Cl^-^ ions inhibits cell division and cell expansion rates during the emergence and development of new leaves and root systems, as well as disturbs the photosynthesis and carbon assimilation [2, 31]. The development of IR28 seedlings was significantly inhibited under salt stress treatment, as demonstrated by a reduction in shoot length (24.4%), root length (11.3%), dry weight (34.2%), and total chlorophyll content (50%) as well as an increase in the number of wilted and rolled leaves (Fig 1A, 1D and 1E). Salt stress, however, caused less damage to DP seedlings than to their counterparts following a 7-day salt stress treatment (Fig 1). Salt-stressed DP seedlings remained healthy, with all their leaves appearing to be dark green in color, in spite of 14.1 and 1.2% reductions in shoot and root length, respectively. This evidence clearly demonstrates that DP seedlings have a higher tolerance for salt stress than IR28 seedlings.

Next, a comparative miRNA sequencing analysis was conducted on DP and IR28 seedlings cultivated under control and salt stress conditions. Eight miRNA sequencing libraries were generated, with the DP and IR libraries returning approximately 37 million and 42 million of the total clean reads, respectively. Comparative analysis identified a total of 69 miRNAs from 31 gene families that are differently expressed in response to salt stress. The diversity and population size of DEmiRNAs in DP and IR28 seedlings were greater in the shoot tissues than in the root tissues (S3 Table). Furthermore, a high number of miRNAs were found to be downregulated in the shoot tissues of DP and IR28 seedlings, accounting for approximately 90 and 81% of the shoot-specific miRNAs, respectively. In contrast, a greater number of miRNAs with increased transcript abundance were identified in the root tissues of DP (46%) and IR28 (58%) seedlings. The large population of DEmiRNAs implies that considerable alterations in the expression of downstream genes and stress-related pathways have been directed by miRNAs at post-transcriptional level.

Seven gene families were found to have a similar trend of expression in both DP and IR28 cultivars under 150 mM NaCl treatment, including *MIR160*, *MIR162*, *MIR166*, *MIR169*, *MIR172*, *MIR528*, and *MIR529* (Fig 3A) Particularly, it has been found that expression of the *MIR160*, *MIR162*, *MIR166*, *MIR172*, and *MIR528* families was downregulated in the shoot tissues of DP and IR28 seedlings under salt stress. In salt-stressed root tissues, the expression of the *MIR160*, *MIR162*, and *MIR528* families was considerably dropped, whereas the expression of the *MIR172* family and the majority of *MIR166* family members were significantly elevated. In the *MIR169* and *MIR529* families, the expression of each member was either increased or decreased across both tissues when exposed to 150 mM NaCl stress. KEGG enrichment analysis revealed that these miRNAs are involved in the miRNA-mediated pathways of ubiquinone, terpenoid-quinone biosynthesis, ascorbate and aldarate metabolism, N-glycan biosynthesis, the MAPK signaling pathway, and ABC transporter and metabolic activities (Fig 3C). In addition, they have been repeatedly reported as part of an evolutionarily conserved salt-stress-responsive system in several plant species [22, 29, 32–34]. For example, miR169 and miR172 are key regulators of salt tolerance in *Arabidopsis*, cereals, and soybeans [22, 29]. An increase in the transcript abundance of miR169 and miR172 results in the inhibition of the NTFY and AP2/ERF transcription factors, respectively, and transcriptional activation of enzymatic ROS scavenger (PEROXIDASES, CATALASES, and SUPEROXIDE DISMUTASES) encoding genes, hence maintaining redox homeostasis and conferring salt tolerance to plants [22, 29, 32, 33]. MiR160 and miR166 have been found as regulators of the AUXIN RESPONSE FACTORS (ARF) and HD-ZIP III transcription factors in *Arabidopsis*, rice, tobacco, and tomato, respectively [35, 36]. They are cable of establishing a cross-talk regulation of auxin and abscisic acid (ABA) signaling pathways to regulate the development of leaves (length and width of leaf blades) and roots (length of roots and number of lateral roots) in response to salt stress. It is believed that this mechanism is part of the plants’ strategy for maintaining osmotic homeostasis and vegetative development under salt stress [36, 37]. In addition, most of these miRNAs have been found to have a similar trend of expression as their counterparts from the sequencing data obtained from rice (cultivars Pusa Basmatti and Pokkali) and green foxtail millet, *Setaria viridis* cultivars A10 and Me034V (S4 Table) [7, 20]. Together, these data have suggested the existence of common miRNA-mediated stress response mechanisms in plants, including DP and IR28 cultivars.

Comparative analysis of miRNA sequencing data from shoot and root tissues of two contrasting rice cultivars detected 18 miRNAs in DP seedlings with distinct expression patterns in response to salt stress treatment. They are members of the *MIR156* (miR156a/b/c/d/e/f/g/h/i/j, miR156g-3p and miR156l), *MIR164* (miR164d), *MIR167* (miR167e/i-3p), *MIR168* (miR168a), *MIR171* (miR171h), *MIR396* (miR396g), *MIR398* (miR398a), *MIR1432* (miR1432), *MIR1846* (miR1846d-5p and miR1846d-3p), *MIR1857* (miR1857-5p and miR1857-3p), *MIR1861* (miR1861a/b/f/g/i/o), *MIR3979* (miR3979-5p and miR3979-3p), and *MIR5508* (miR5508) gene families. In response to salt stress, the expression of these miRNAs is considerably altered in DP seedlings. Whereas, their expression in IR28 seedlings was either negligible or displayed an opposite pattern from that of their counterparts in DP seedlings. In addition, they have been found to be involved in the regulation of a range of developmental and physiological processes in many plant species, such as transcriptional regulation, osmotic adjustment, root development, ROS scavenger production, auxin and ABA signaling regulation, and functional protein synthesis. Therefore, these miRNA are likely to be responsible for the higher level of salt tolerance in DP seedlings [2, 13, 38].

In plants, miRNA-mediated transcriptional regulation plays a critical role in controlling various biological and stress response processes. The NAC transcription factor family is one of the most highly conserved transcription factor families and regulates numerous stress-responsive genes, including those associated with salt stress [15]. In this study, the abundance of miR164d transcript, a regulator of NAC21/22 transcription factor, was found to be reduced by 1.7- and 8.5-fold in the shoot and root tissues of salt-stressed DP seedlings, respectively. Similar to miR164d in DP seedlings, the transcript abundance of the corresponding miR164 sRNAs was also observed to be significantly reduced in salt-stressed rice (cultivars Pusa Basmatti and Pokkali) and *S*. *viridis* Me034V (S4 Table). In addition, the reduction in miR164d transcript in DP seedlings was experimentally validated to result in a 1.5- and 4.9-fold increase in the expression of NAC21/22 transcription factor in the shoot and root tissues, respectively (Fig 4A). Overexpression of NAC encoding genes has been reported to markedly enhance the increased level of compatible solutes synthesized in the root tissues, such as soluble sugars, glycine betaine, and free proline, in genetically modified *Arabidopsis* [39], rice cultivars Yuanfengzao and Nipponbare [40, 41], soybeans [42], and maize [43]. This mechanism aids in the maintenance of osmolality and stabilization of functional proteins in plants exposed to salt stress [40, 42].

The *MIR168* and *MIR1857* gene families have also been discovered as regulators of transcriptional processes in plants. MiR168a was identified as a negative regulator that controls the expression of genes encoding AGO1, a slicer protein in the catalytic core of the RISC complex that catalyzes miRNA-directed cleavage of transcribed genes [44]. AGO1 is also involved in the growth phase transition and salt stress response systems in plants [44, 45]. In salt-stressed *Arabidopsis*, the levels of miR168a and AGO1 showed an opposing expression pattern, with the former displaying a lower expression than in the non-stressed plants. In this study, a similar expression trend was observed for miR168a, with its abundance decreasing by 1.5- and 0.2-fold in the shoot and root tissues of salt-stressed DP seedlings, respectively.

MiR1857 sRNAs regulate transcriptional processes via targeting the conserved PLANT HOMEODOMAIN (PHD) FINGER proteins, a small zinc-coordinating domain located in the nucleus [46]. Downregulation of miR1857 sRNAs has led to an increased degree of salt stress tolerance in maize, tomato, and *Brassica rapa* via transcriptional regulation of downstream genes involved in the ABA signaling pathway by either modulating the transcriptional process or recruiting co-activators and co-repressors [46–48]. In this study, miRNA sequencing data and enrichment analyses revealed that miR1857 targets PHD finger proteins in both DP and IR28 seedlings. The transcript abundance of miR1857 sRNA showed a substantial reduction of up to 6.0-fold in salt-stressed DP seedlings, whereas it remained unchanged in salt-stressed IR28 seedlings. These results suggest that miR164d, miR168 and miR1857 sRNAs play an important role in the enhancement of salt stress adaptation in DP seedlings via regulating plant transcriptional processes.

Regulation of root development is another effective strategy that plants employ to cope with salt stress [49]. MiRNA sequencing data revealed a significant increase in the abundance of miR171h (1.7-fold in shoots and 1.1-fold in roots) and miR396g (3.1-fold in roots), and a significant decrease in the abundance of miR167e/i-3p (-2.2-fold in roots) in salt-stressed DP seedlings. Previous research has shown that the upregulation of miR171 sRNAs enhances primary root elongation via manipulation of the quiescent center as well as the expression of Scarecrow-like 6-II (SCL6-II), SCL6-III, and SCL6-IV encoding genes in *Arabidopsis*, wheat, cotton, and rice cultivar Pokkali [7, 50, 51]. Similarly, an increase in the miR396 transcript abundance has also been identified to promote primary root growth in *Arabidopsis* and *Medicago truncatula*. MiR396 has been identified to target the GROWTH REGULATING FACTORS (GRFs), and by inhibiting the expression of GRF encoding genes, miR396 enhances the division of plant root cells [52, 53]. Whereas miR167 sRNAs are negative regulators of ARF6-like and ARF8-like transcription factors in *Arabidopsis*, rice cultivar IR64, and maize [54–56]. The downregulation of miR167 sRNAs led to an increase in the formation of adventitious roots in stressed plants. Collectively, the expression of miR167e, miR167i, miR171h, and miR396g in salt-stressed DP seedlings could, in part, explain the similarities in the development of root systems observed in DP seedlings under control and salt-stress treatments.

Plants also produce a variety of ROS scavengers to counter oxidative stress derived from the excessive accumulation of ROS species under salt stress [57, 58]. In this study, the transcript abundance of miR156 and miR398a sRNAs, the endogenous miRNAs that control the synthesis of ROS scavengers, anthocyanin and CSD2, were found to be upregulated by up to 1.0- and 2.8-fold in the root tissues of salt-stressed DP seedlings, respectively. Increased abundance of miR156 sRNAs has been reported to enhance salt stress tolerance in *Arabidopsis*, alfalfa, pear, and apple [57–60]. In response to salt stress, miR156 sRNAs form a module with their target SPL proteins (miR156-SPL) to perceive the external stimuli and increase salt stress tolerance via inducing an appropriate accumulation of anthocyanin in plants [60]. It is also worth noting that the increase in miR156 sRNA transcript abundance at a relatively low level is more beneficial for the vegetative and reproductive development of plants, such as increased tillering and biomass yield. Moderate and high levels of miR156 sRNA, in contrast, have been found to cause dwarfism, delayed flowering, and reduced biomass in genetically modified plants [61, 62]. Similarly, an increased abundance of miR398a was found to gradually accumulate in *Arabidopsis* and poplar subjected to prolonged (over 72 hours) salt stress exposure [63, 64]. MiR398 sRNAs direct the biosynthesis of CSDs, the antioxidant enzymes that are used to convert highly toxic superoxide (O_2_^-^) ions to less toxic hydroperoxide (H_2_O_2_), thereby reducing the level of oxidative stress in plants [63, 64].

Other miRNAs that are specifically expressed in salt-stressed DP seedlings have been identified to regulate genes involved in various processes in plants, such as vegetative development and abiotic stress tolerance (miR3979-3p, miR3979-5p, and miR1861), translation regulation (miR1846a), signal transduction pathway (miR1432), and carotenoid biosynthesis (miR1857-3p).

## Conclusion

Comparative analysis of the DP and IR28 miRNA sequencing data detected 69 DEmiRNAs in response to salt stress treatment. Among them, 18 miRNAs from 13 miRNA gene families (*MIR156*, *MIR164*, *MIR167*, *MIR168*, *MIR171*, *MIR396*, *MIR398*, *MIR1432*, *MIR1846*, *MIR1857*, *MIR1861*, *MIR3979*, and *MIR5508*) were identified as being specifically expressed in salt-tolerant DP seedlings. Together with miRNA sequencing data from the other rice cultivars and *S*. *viridis*, our results suggest that these miRNAs enhance the salt tolerance of DP seedlings by regulating a range of target genes involved in different stress responsive mechanisms, such as transcriptional regulation, osmotic adjustment, root development, ROS scavenger production, auxin and ABA signaling regulation, and other functional protein synthesis. However, more research is required to reach a more conclusive argument regarding the contributions of these miRNAs to salt stress tolerance in rice.

## Supporting information

S1 Fig Predicted miR164d, miR169i, mir172d, miR398a, miR528, and miR529b target site positions on the *NAC21/22*, *NTYF*, *AP2/ERF*, *CSD2*, *AO* and *SPL14* genes determined by the psRNATarget tool, respectively. Double dots represent Watson-Crick base pairs and single dot represents G:U wobble base pairs.
(DOCX)

S1 Table List of primer used for miRNA-specific cDNA synthesis primers and RT-qPCR analysis. (DOCX)

S2 Table Expression of identified miRNAs. (XLSX)

S3 Table List of DEmiRNAs. (XLSX)

S4 Table Comparison of mRNA expression. (XLSX)
