# Supplementary material for: Comparative analysis of microRNA expression profiles in shoot and root tissues of contrasting rice cultivars (Oryza sativa L.) with different salt stress tolerance
Source: PLoS One. 2023 May 24;18(5):e0286140. doi: 10.1371/journal.pone.0286140 (PMC10208480; doi:10.1371/journal.pone.0286140)
Supplement: S1 Fig — Double dots represent Watson-Crick base pairs and single dot represents G:U wobble base pairs. (DOCX) [file pone.0286140.s001.docx]

**Supplementary Material**

**Comparative analysis of microRNA expression profiles in shoot and root tissues of contrasting rice cultivars (*Oryza sativa* L.) with different salt stress tolerance**

Duc Quan Nguyen^1^, Ngoc Lan Nguyen^1^, Van Tung Nguyen^1^, Thi Huong Giang Tran^1^, Thanh Hien Nguyen^1^, Thi Kim Lien Nguyen^1^ and Huy Hoang Nguyen^1,2,*^

*^1^ Institute of Genome Research - Vietnam Academy of Science and Technology, 18 Hoang Quoc Viet, Cau Giay, Hanoi, 10000, Vietnam*.

*^2^ Graduate University of Science and Technology, Vietnam Academy of Science and Technology, 18 Hoang Quoc Viet, Cau Giay, Hanoi 100000, Vietnam*.

******* *Corresponding author:*

Email: [nhhoang@igr.ac.vn](mailto:nhhoang@igr.ac.vn)


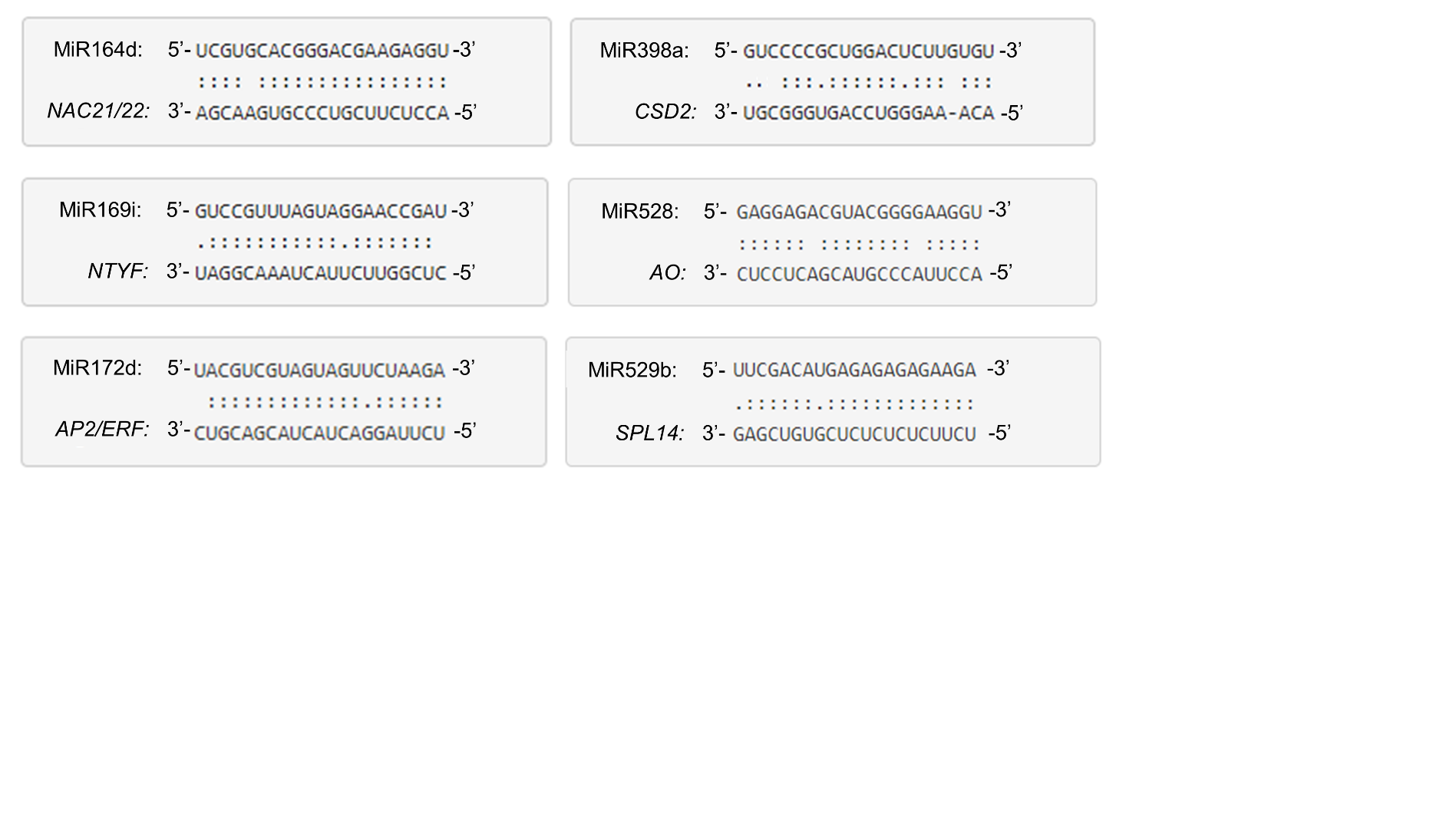


**S1 Fig:** **Predicted miR164d, miR169i, mir172d, miR398a, miR528, and miR529b target site positions on the *NAC21/22*, *NTYF*, *AP2/ERF*, *CSD2, AO* and *SPL14* genes determined by the psRNATarget tool, respectively**. Double dots represent Watson-Crick base pairs and single dot represents G:U wobble base pairs.
