# Supplementary material for: Comparative analysis of microRNA expression profiles in shoot and root tissues of contrasting rice cultivars (Oryza sativa L.) with different salt stress tolerance
Source: PLoS One. 2023 May 24;18(5):e0286140. doi: 10.1371/journal.pone.0286140 (PMC10208480; doi:10.1371/journal.pone.0286140)
Supplement: S1 Table — (DOCX) [file pone.0286140.s002.docx]

**Supplementary Material**

**Comparative analysis of microRNA expression profiles in shoot and root tissues of contrasting rice cultivars (*Oryza sativa* L.) with different salt stress tolerance**

Duc Quan Nguyen^1^, Ngoc Lan Nguyen^1^, Van Tung Nguyen^1^, Thi Huong Giang Tran^1^, Thanh Hien Nguyen^1^, Thi Kim Lien Nguyen^1^ and Huy Hoang Nguyen^1,2,*^

*^1^ Institute of Genome Research - Vietnam Academy of Science and Technology, 18 Hoang Quoc Viet, Cau Giay, Hanoi, 10000, Vietnam*.

*^2^ Graduate University of Science and Technology, Vietnam Academy of Science and Technology, 18 Hoang Quoc Viet, Cau Giay, Hanoi 100000, Vietnam*.

********Corresponding author:*

Email: [nhhoang@igr.ac.vn](mailto:nhhoang@igr.ac.vn)

**S1 Table: List of primer used for miRNA-specific cDNA synthesis primers and RT-qPCR analysis.**

| **Primer name** | **Sequence (5'-3')** | **cDNA amplicon** | **gDNA amplicon** |
| --- | --- | --- | --- |
| *MiRNA-specific cDNA synthesis primers* | | | |
| pOsmiR164dSL-RT | GTCGTATCCAGTGCAGGGTCCGAGGTATTCGCACTGGATACGACAGCACG |  |  |
| pOsmiR169rSL-RT | GTCGTATCCAGTGCAGGGTCCGAGGTATTCGCACTGGATACGACCAGGCA |  |  |
| pOsmiR172dSL-RT | GTCGTATCCAGTGCAGGGTCCGAGGTATTCGCACTGGATACGACATGCAG |  |  |
| pOsmiR396gSL-RT | GTCGTATCCAGTGCAGGGTCCGAGGTATTCGCACTGGATACGACCCGTTC |  |  |
| pOsmiR398aSL-RT | GTCGTATCCAGTGCAGGGTCCGAGGTATTCGCACTGGATACGACAAGGGG |  |  |
| pOsmiR529bSL-RT | GTCGTATCCAGTGCAGGGTCCGAGGTATTCGCACTGGATACGACAAGCTG |  |  |
| U6SL-RT | GTGCAGGGTCCGAGGTTTTGGACCATTTCTCGAT |  |  |
| *RT-qPCR primers* | | | |
| pOsmiR164d | F: AACAGTGTGGAGAAGCAGGG | 66 bp | 66 bp |
|  | R: CCAGTGCAGGGTCCGAGGTA |  |  |
| NAC21/22 | F: ATTGCTCGTCGGTATGCGAA | 148 bp | n/a |
|  | R: ACACAAGACCCAGTCCTCCT |  |  |
| pOsmiR169r | F: AACACGCTAGCCAAGGATGA | 66 bp | 66 bp |
|  | R: CCAGTGCAGGGTCCGAGGTA |  |  |
| NTFY | F: AGACAGCTCCGTGCAAAGTT | 75 bp | 251 bp |
|  | R: CCGGGATTCATGGAGGTACG |  |  |
| pOsmiR172d | F: AGCCAGCGAGAATCTTGATGA | 67 bp | 67 bp |
|  | R: CCAGTGCAGGGTCCGAGGTA |  |  |
| AP2/ERF | F: ATTCGACAGCGAAGTAGAGGC | 85 bp | 260 bp |
|  | R: CTCGAAGTTGGTGACGGCTT |  |  |
| pOsmiR398a | F: AACACGCTGTGTTCTCAGGT | 66 bp | 66 bp |
|  | R: CCAGTGCAGGGTCCGAGGTA |  |  |
| CSD2 | F: CCTCTGTGACGGGAAGTGTC | 80 bp | 80 bp |
|  | R: ATTAGTGGTGTCACCGAGCG |  |  |
| ACT | F: CAGCCACACTGTCCCCATCTA | 67 bp | 67 bp |
|  | R: AGCAAGGTCGAGACGAAGGA |  |  |
| UBQ | F: TGGTCAGTAATCAGCCAGTTTGG | 81 bp | 81 bp |
|  | R: GCACCACAAATACTTGACGAACAG |  |  |
| U6 | F: GGAACGATACAGAGAAGATTAGCA | 78 bp | 78 bp |
|  | R: GTGCAGGGTCCGAGGT |  |  |
